# Can CRISPR/Cas Technology Be a Felicitous Stratagem Against the COVID-19 Fiasco? Prospects and Hitches

**DOI:** 10.3389/fmolb.2020.557377

**Published:** 2020-09-10

**Authors:** Rocktotpal Konwarh

**Affiliations:** ^1^Department of Biotechnology, Addis Ababa Science and Technology University, Addis Ababa, Ethiopia; ^2^Centre of Excellence-Nanotechnology, Addis Ababa Science and Technology University, Addis Ababa, Ethiopia

**Keywords:** COVID-19, SARS-CoV-2, CRISPR/Cas, diagnostics, viral inactivation, RNA

## Abstract

The current global debacle of COVID-19, spelled by SARS-CoV-2 needs no elaboration. With incessant and constantly clambering number of deaths across various nations, the need of the hour is to develop readily deployable, fast, affordable detection assays and kits, yielding precise and consistent results as well as timely availability of efficacious anti-SARS-CoV-2 strategies to contain it. Conventionally employed real time PCR based technique for detection of the virus suffers from a couple of handicaps. Amongst other approaches, CRISPR based technology has ushered in new hopes. Recent efforts have been directed toward developing CRISPR/Cas based low-cost, rapid detection methods as well as development of one-pot assay platforms. The plausible application of CRISPR-Cas system to counteract the viral assault has also been assessed. The write up in this article mirrors the current status, the prospects and the practical snags of CRISPR/Cas technology for the detection and inactivation of the novel corona virus, SARS-CoV-2.

## Introduction

The constantly escalating number of articles in various scientific literature repositories attests the continual progress in the explication of the virology of SARS-CoV-2 and the pathogenesis of COVID-19 (Chen et al., 2020; Li et al., 2020b; Monteil et al., 2020; Rabi et al., 2020; Wan et al., 2020; Wu, 2020; Xie and Chen, 2020). Various tactics for restricting the viral fusion/entry, interruption of the viral replication machinery, and suppression of inflammatory response, use of neutralizing antibodies including nanobodies and convalescent plasma treatment, besides the ongoing efforts and challenges in developing vaccines against SARS-CoV-2 are being appraised in a progressive context (Jiang et al., 2020; Konwarh, 2020; Li et al., 2020a). Howbeit, with the persistently increasing COVID-19 cases in various countries (World Health Organization, 2020), it becomes imperative to bring the detection-tests ‘closer’ to the patients and clinicians, particularly in terms of the cost, rapidity and ease of use. One may easily perceive the requisite to ramp up testing at a rapid pace in countries where the death toll is alarmingly high (Médecins Sans Frontières, 2020). By the same token, media reports vouch for the criticism, faced by different countries for the pitiable mass testing proficiency, bracketed together with scarcity of COVID-19 testing kits (Al Jazeera and News Agencies, 2020). On the other hand, asymptomatic transmission of the virus seems to be the Achilles’ heel amidst the current control strategies of the pandemic (Day, 2020; Gandhi et al., 2020). This further necessitates the extension and scale-up of SARS-CoV-2 assessment. Needless to stress that ascertaining foci of infections as well as reduction in false negative rates and elimination of ambiguity in results demand timely, pre-emptive and regular testing.

Pertinently, exploiting CRISPR/Cas (clustered regularly interspaced short palindromic repeats/CRISPR-associated) systems (Makarova et al., 2020) for sensitive nucleotide detection has been an interesting endeavor in recent years (Jia et al., 2020). Resorting to CRISPR/Cas systems for the diagnosis as well as inactivation of RNA and DNA viruses has opened up new portals in clinical virology (Jia et al., 2020). These systems employ platforms like specific high sensitivity enzymatic reporter unlocking or SHERLOCK (Gootenberg et al., 2017), DNA endonuclease-targeted CRISPR *trans* reporter or DETECTR (Chen et al., 2018), 1-h low-cost multipurpose highly efficient system or HOLMES (Li et al., 2019) and Cas13-assisted restriction of viral expression and readout or CARVER (Freije et al., 2019). To cite for evidence, SHERLOCK in combination with HUDSON (heating un-extracted diagnostic samples to obliterate nucleases) has been used directly to detect Zika and Dengue viruses from patients’ bodily fluids (Myhrvold et al., 2018). Such platforms are also being harnessed in the battle against SARS-CoV-2. These are expected to ease the burden placed on manufacturing potential for the costly conventional RT-qPCR testing reagents and consumables, dedicated instrumentation, and the absolute requisite of trained laboratory personnel that hinders widespread testing. However, it should be borne in mind that the various nucleic acid based detection-approaches like PCR, isothermal nucleic acid amplification-based methods, CRISPR/Cas platforms as well as immunoassay based point-of-care lateral flow tests are marked with their corresponding pros and cons (Bai et al., 2020; Shen et al., 2020). Having said that, let us flick through some of the recent global endeavors in harnessing CRISPR/Cas technology to detect and target SARS-CoV-2 in the following section.

## Prospects of CRISPR/Cas Systems for Detection and Inactivation of SARS-CoV-2

In the battle against the novel coronavirus, Sherlock Biosciences and Mammoth Biosciences have come up with innovative CRISPR platforms for COVID-19 diagnosis, facilitating visual readout. SHERLOCK platform targets two SARS-CoV-2 genes – the *S* gene and *Orf1ab* while Mammoth Biosciences’ platform DETECTR targets the *N* and *E* viral genes (Prabhune, 2020). In a recent study, an inexpensive, fast (<40 min) and precise CRISPR-Cas12 based lateral flow assay- SARS-CoV-2 DETECTR for diagnosis of SARS-CoV-2 was reported (Broughton et al., 2020). The researchers had resorted to the use of contrived reference samples and clinical samples from infected US patients to attest the assay-performance in lines with the US CDC SARS-CoV-2 RT-PCR analysis. The protocol encompassed concomitant reverse transcription and isothermal amplification using loop-mediated amplification (RT-LAMP) from RNA, sampled from nasopharyngeal or oropharyngeal swabs in universal transport media (UTM). Cas12 assisted unmasking of predefined coronavirus sequences was followed by the cleavage of a reporter molecule, confirming the eventual detection of the virus. On a similar note, researchers from Argentina and CASPR Biotech, United States have described a CRISPR/Cas12 based ultrasensitive, fast and portable SARS-CoV-2 detection (Lucia et al., 2020). The suitability of using saliva specimens for diagnosis of COVID-19 was stressed upon in the report. Naturally occurring molecules in saliva did not seem to exert any inhibitory influence in the CRISPR based paper strip assay. With an objective to ensure an unambiguous detection of SARS-CoV-2, a cross-institutional work in China encompassed the designing of CRISPR RNAs (crRNAs) with the projected potency to discern single nucleotide polymorphisms (SNPs) from other SARS or SARS-related viruses, on the four domains of the *orf1a*, *orf1b*, *N* and *E* genes over the Wuhan-Hu-1 strain (GenBank accession number MN908947) (Wang et al., 2020). Secondary structure and spacer sequence of the crRNA dictated the crRNA-dependent precise scissoring efficacy, eventually affecting the readout signal. Based on the sensitivity, indicated by fluorescence intensity, *E*-crRNAmix (against the target *E* gene) was employed in the screening of SARS-CoV-2 while *orf1a*-crRNAmix, *orf1b*-crRNAmix, *N*-crRNAmix were employed for additional confirmatory diagnosis. The authors validated their CRISPR/Cas12a-based- detection with naked eye readout (CRISPR/Cas12a-NER) assay (with an assay time of 15 min) in clinical diagnosis of COVID-19, demonstrating 100% agreement with qPCR assays, with the kappa value (κ) being 1.0 (*P* < 0.001).

On the other hand, a prospective CRISPR-novel Corona Virus (CRISPR-nCoV) test based on the concert of a recombinase polymerase amplification (RPA) step, subsequent T7 transcription and Cas13 detection step had been documented. With a near single-copy sensitivity and shorter turn-around time than RT-PCR, the approach demonstrated a cent percent clinical sensitivity in detecting 52 COVID-19 positive cases [confirmed by metagenomic next-generation sequencing (mNGS)], without any false positives for the 62 tested negative cases (Hou et al., 2020). Similarly, Metsky et al. (2020) had recently evaluated genomically-comprehensive machine learning SARS-CoV-2 assay designs based on CRISPR/Cas13 detection approach, incorporating synthetic targets with fluorescent and lateral flow detection. The detection of synthetic SARS-CoV-2 RNA was reported at 10 copies per microliter. However, assessing the sensitivity of the assay for clinical isolates and patient samples as well as demonstrating the specificity at species and subspecies levels of highly related viruses demand attention. In a similar vein, Rauch et al. (2020) have recently proposed the application of CREST (Cas13-based, Rugged, Equitable, Scalable Testing) protocol as an orthogonal solution to augment testing potency for SARS-CoV-2. The approach involves the use of simple, easy to operate, portable thermocyclers and plastic-filter-based LED visualizers. The feasibility of documenting results with a smartphone camera and consequent uploading to the cloud is expected to facilitate point-of-care testing (POCT). At this juncture, it would be easily graspable that highly multiplexed CRISPR-based nucleic acid detection systems with feasibility to scale and miniaturize have the potency to shift diagnostic and surveillance endeavors from directed assessment of high-priority samples to all-inclusive testing of large sample groups. This would aid both patients and public health care practitioners. In this context, Ackerman et al. (2020)’s report evinced the fruition of a Combinatorial Arrayed Reactions for Multiplexed Evaluation of Nucleic acids Cas13 (CARMEN-Cas13) based multiplexed assay, capable of concomitant differentiation of 169 human-associated viruses with at least 10 published genome sequences (as on 24, October 2018). Besides efficient subtyping of influenza A strains and multiplexed discerning of HIV drug-resistance mutations, the platform was adapted promptly to slot in an additional crRNA for the detection of SARS-CoV-2. In fact, the authors have forwarded the possibility of single massive-capacity chip (mChip) -based parallel testing of 400 + samples against their coronavirus panel. By the same token, use of the mChip is expected to considerably cut down the reagent cost per test corresponding to standard multiwell-plate SHERLOCK tests, besides curtailing the number of protocol-steps and overall execution-time.

**FIGURE 1:**
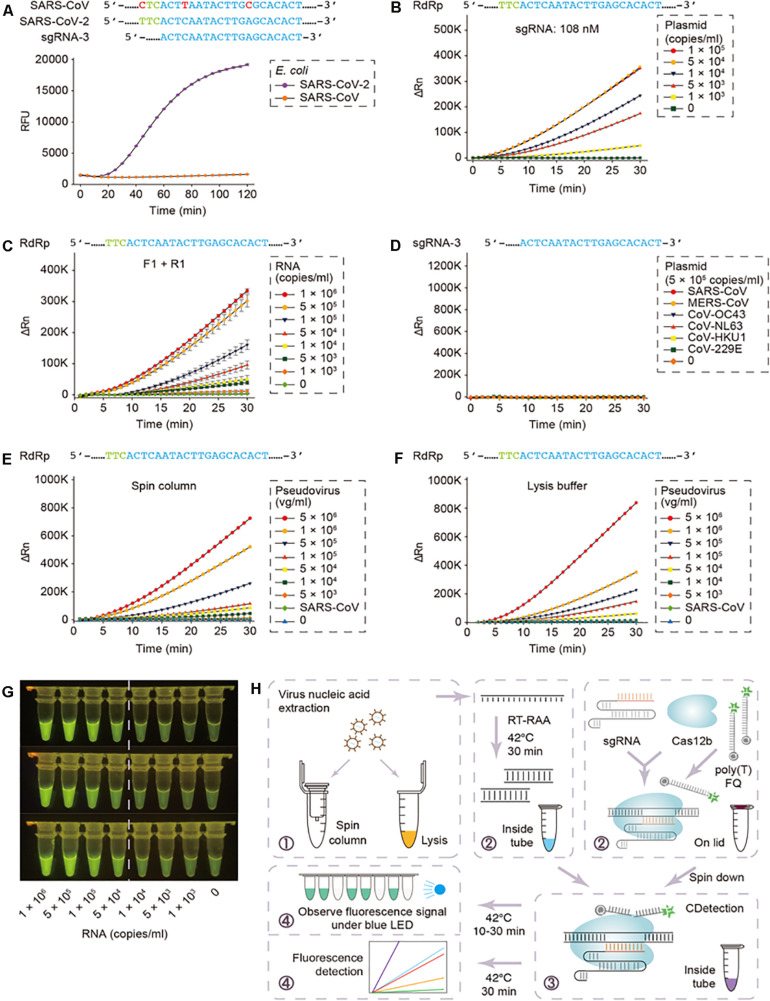
CASdetec used for SARS-CoV-2 detection. **(A)** Fluorescence kinetics of sgRNA-3 for RdRp detection. *E. coli* cells bearing Blunt-SARS-CoV-RdRp or Blunt-SARS-CoV-2-RdRp were pre-incubated at 95 °C for 10 min and used as templates for RAA and CDetection. PAM sequences are colored in green, proto-spacers are colored in blue, base pair mismatches are colored in red. Error bars indicate standard errors of the mean (SEM), *n* = 3. RFU, relative fluorescence units. **(B)** Fluorescence kinetics of RdRp detection using 108 nM sgRNA-3. Plasmid bearing SARS-CoV-2-RdRp was serially diluted as shown in the legend. *n* = 2. ΔRn, ΔFluorescence, which refers to the Rn value of an experimental reaction minus the Rn value of the baseline signal generated by ABI 7500. **(C)** Fluorescence kinetics of F1- and R1-based RdRp detection. SARS-CoV-2-RdRp RNA was serially diluted as shown in the legend. Error bars indicate (SEM), *n* = 3. **(D)** Evaluation of cross-reactivity. Plamids containing target RdRp region from six human epidemic coronaviruses were serially diluted as the shown in the legend. *n* = 2. **(E)** Detection of SARS-CoV-2 pseudovirus. Virus genome was extracted using the virus RNA extraction kit (spin column). SARS-CoV were diluted to 5 × 10^5^ copies/mL. *n* = 2. **(F)** Detection of SARS-CoV-2 pseudovirus. Virus was treated by direct lysis. SARS-CoV was diluted to 5 × 10^5^ copies/mL. *n* = 2. **(G)** CASdetec results could be directly observed under blue LED. 3 replicates of products from **(C)** were imaged upon blue LED illumination. **(H)** Schematics showing the workflow of CASdetec. Virus genome was extracted by kit or direct lysis. Target sequences were pre-amplified by isothermal amplification, followed was CDetection. Fluorescence signals were obtained either from fluorescence reader or direct observation under blue light (Reproduced from Guo et al., 2020, under the provisions of Creative Commons Attribution 4.0 International License, http://creativecommons.org/licenses/by/4.0/, Copyright© The Author(s) 2020).

Although, these assays seem promising, the practical snags associated with the current CRISPR-based nucleic acid detection methods include the requisites of distinct nucleic acid amplification in various reaction platforms and numerous manual operations. The latter obfuscates the assessment protocols besides adding to the trouble of carry-over adulterations, attributed to the transfer of amplification products. In this context, Ding et al. (2020) from University of Connecticut Health Center have come up with an ‘All-In-One Dual CRISPR-Cas12a’ (termed ‘AIOD-CRISPR’) assay for low-cost, fast (typically 5–20 min), ultrasensitive, precise and visual detection of nucleic acid to assist clinicians. The various components for isothermal amplification and CRISPR-based detection can be prepared in a one-pot format, thereby, simplifying the detection procedures and abolishing the carry-over contamination-risks. The AIOD-CRISPR assay method could identify nucleic acids (DNA and RNA) of the SARS-CoV-2 and HIV-1 with a sensitivity of a few copies. Furthermore, a comparable sensitivity with RT-PCR was documented while detecting HIV-1 RNA of human plasma samples within less than 20 min. Integrating the AIOD-CRISPR assay into microfluidic diagnostic chip could result in realizing a simple, rapid and affordable point-of-care diagnostic platform for SARS-CoV-2 detection as well. By the same token, Guo et al. (2020) had coalesced sample treatment strategies and nucleic-acid amplification protocols to a previously reported Cas12b-mediated DNA detection (CDetection) (Teng et al., 2019), leading to the establishment of a CASdetec (CRISPR assisted detection) platform, exhibiting a detection limit of 1 × 10^4^ copies/mL for SARS-CoV-2 pseudovirus, without any cross-reactivity (Figure 1). The researchers employed a 7 nt poly-T fluorescence quencher and a single guide RNA-3, capable of discerning between the closely related SARS-CoV-2 and SARS-CoV, besides exhibiting the most distinct fluorescence signal. A hallmark feature of this study was the concurrent execution of the RT-recombinase aided amplification (RT-RAA) and CDetection within a single tube without lid-opening, thereby, lowering the risk of plausible aerosol contamination and associated false positives.

The afore-stated citations assuredly testify the immense prospects of harnessing CRISPR/Cas systems in the SARS-CoV-2 detection. As noted previously, companies like Mammoth Biosciences, Sherlock Biosciences and CASPR Biotech are currently devoting substantial time and resources to commercialize their respective CRISPR based diagnostic assays. Pertinently, under ‘emergency use’ provisions, the US Food and Drug Administration’s (FDA) emergency-use authority has recently approved a CRISPR-based diagnostic kit developed by Sherlock Biosciences to allay the COVID-19 detection backlogs in the US (Satyanarayana, 2020). Similarly, in the Indian perspective, the Council of Scientific and Industrial Research’s (CSIR) component lab, Institute of Genomics and Integrative Biology (CSIR-IGIB) and TATA Sons have signed an MoU for licensing of ‘KNOWHOW’ for CRISPR based FNCAS9 Editor Linked Uniform Detection Assay (*FELUDA*) to cater to mass testing in a prompt manner (Sarkar, 2020).

**FIGURE 2:**
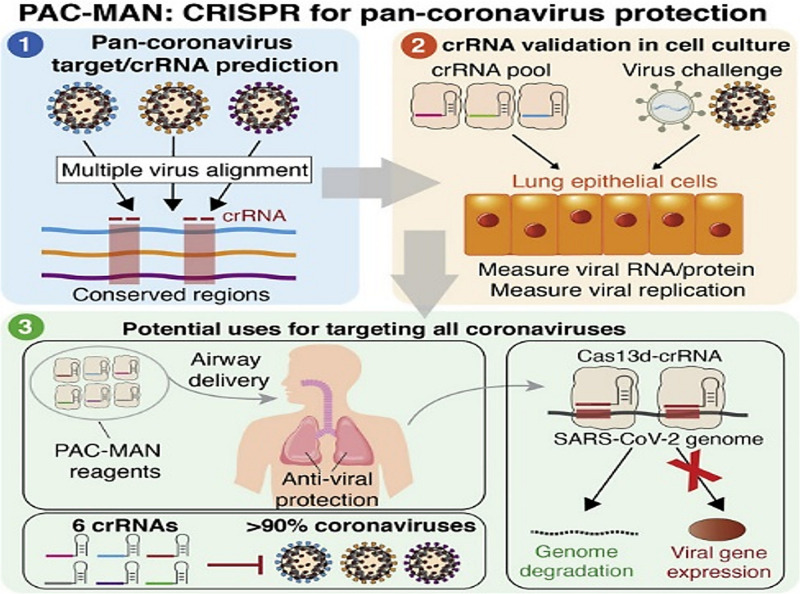
Highlights of PAC-MAN based strategy to target conserved sequences across coronaviruses and other pathogenic viruses (Reproduced from Abbott et al., 2020, based on the reuse-provisions of Elsevier’s COVID-19 Resource Centre, Copyright© 2020 Elsevier, Inc.).

Deploying CRISPR/Cas system to combat viral assault seems to be another interesting proposition. Targeting the positive sense genome and viral mRNAs for concomitantly cleaving viral genome templates and shutting off their gene expression would be a stringent way to diminish the replication of SARS-CoV-2. In an earlier creditable attempt to harness Cas13’s tailorable RNA-targeting capacity, researchers from Broad Institute of Massachusetts Institute of Technology (MIT) and Harvard, Cambridge, United States reported an end-to-end technology platform, Cas13-assisted restriction of viral expression and readout (CARVER) (Freije et al., 2019). The researchers assessed the efficacy of their programmed Cas13 in targeting three distinct ssRNA viruses- lymphocytic choriomeningitis virus (LCMV), influenza A virus (IAV), and vesicular stomatitis virus (VSV). The programmed Cas13 was robust enough to outshine the actively replicating and evolving viral RNA. Another facet of the study was the non-documentation of crRNA target site mutations (that might decrease the potency of antiviral strategies) following Cas13 treatment over 2-days period. Such multimodal tools are envisaged to permit both visualization and perturbation of viral replication with extremely high precision. A simple amendment of the crRNA sequence might assist in keeping pace with those viruses that plausibly tend to alter its own sequences as a consequence of therapy or in an outbreak. Elucidation of the precise action and efficacy of the tool for one virus is expected to enable the facile designing of sequences for various other viruses including SARS-CoV-2. Recently, considerable computational analysis and experimentations were put forth in a study streamlined in repurposing the RNA-guided RNA endonuclease activity of Cas13d in mammalian cells against SARS-CoV-2 and live influenza A virus (IAV) (Abbott et al., 2020). Screening a panel of crRNAs, targeting conserved viral regions, unveiled a group of six crRNAs that could target 91% of all coronaviruses and a group of 22 crRNAs with potential to target all sequenced coronaviruses with no mismatches. Limited by non-accessibility of widely available laboratory strains, the researchers resorted to synthesized fragments of SARS-CoV-2 (besides using live H1N1 IAV) to evaluate their Prophylactic Antiviral CRISPR in huMAN cells (PAC-MAN) strategy (Abbott et al., 2020). The PAC-MAN strategy was effective enough in degrading the SARS-CoV-2 sequences and live IAV genome in human lung epithelial cells (Figure 2). Pertinently, Cas13d could target conserved genomic regions of SARS-CoV-2, encoding the RNA-dependent RNA polymerase (RdRP) and nucleocapsid proteins that are of paramount importance for coronavirus replication and function.

## Discussion and Conclusion

Post-perusal of the literature, cited in this article, one may easily perceive the prospects of employing CRISPR technology to address the viral assault as well as improve testing speed in the context of global paucity of timely COVID-19 tests. As far as the use of CRISPR technology for viral detection is concerned, researchers must address plausible off-target effects of CRISPR which might hinder the precise nucleotide detection (Jia et al., 2020). The accuracy may also be affected as CRISPR/Cas effectors tend to show some sort of tolerance to mismatches between the guide RNA and target nucleotides. Furthermore, previous reports have evinced the failure of CRISPR to detect target DNA if the amplification product is < 1–10 nm within the reaction mix (Teng et al., 2019). In this context, strategies to augment the molecular collisions between CRISPR and target (e.g., via enhancing the sgRNA concentrations, as exemplified in the work by Guo et al., 2020) could be instrumental in increasing reaction-rate as well as amplifying fluorescence-signal and the signal-to-background ratio.

Abbott et al. (2020)’s study testifies the prospects of using CRISPR based systems to inactivate the virus. The researchers, however, did not conduct any animal studies. Ferrying the Cas13 in specific target-zones in a living person (*in vivo* delivery) could be a matter of concern. Although, the PAC-MAN approach (Abbott et al., 2020) may be projected as a ‘potential pan-coronavirus line of attack’ the development of appropriate delivery platforms demands utmost deliberation. Nanobiomaterials may be assessed as prospective contrivance (Chan, 2020). However, the combat zone in case of the coronavirus is the lung- comparatively inaccessible and mucus-packed that might interfere with the directed delivery. A combinatorial approach involving clinically administered drugs would be an interesting proposition to evaluate plausible synergistic viral inactivation. Assessment of the timescale and extent to which viral RNAs might evolve possible resistance to Cas13 targeting also demands attention. However, in the context of limited FDA-approved human trials of CRISPR technology for disease treatment, caution must be maintained *vis-à-vis* possible inflammation and other negative clinical repercussions. Validation of the promising approaches such as PAC-MAN and others using appropriate preclinical animal models or relevant organoid models and eventual clinical trials would obviously be a colossal and arduous task.

At this juncture, it is worthwhile to mention that CRISPR/Cas9 technology has also assisted researchers to generate human angiotensin converting enzyme 2 (hACE2, playing critical role in the viral pathogenesis) knock-in mice (Sun et al., 2020). Remarkably, both young and aged hACE2 mice sustained elevated viral loads in lung, trachea, and brain post-intranasal infection in comparison to wild-type C57BL/6 mice while heightened cytokines level and interstitial pneumonia were documented in SARS-CoV-2 infected aged hACE2 mice. On the other hand, intragastric viral inoculation led to the establishment of infection and subsequent pulmonary pathological alterations in the hACE2 mice. Needless to say, appropriate animal models, recapitulating the clinical progression, evident in COVID-19 patients would support the investigations pertaining to SARS-CoV-2 infection and transmission, besides assisting research-activities to find prospective vaccines and therapeutics.

It is also to be noted that ACE2 receptors are present in key metabolic organs and tissues, including kidneys and the pancreatic β-cells. Besides various autoinflammatory/autoimmune conditions (Rodríguez et al., 2020), a couple of recent observations have also evinced potential diabetogenic effect of COVID-19 (Aloysius et al., 2020; Ren et al., 2020; Rubino et al., 2020). Attributable to the tropism of SARS-CoV-2 for the pancreatic β-cells and impairment of their function in concert with the cytokine storm and counter-regulatory hormonal responses, new-onset diabetes besides severe metabolic complications of preexisting diabetes, including diabetic ketoacidosis and hyperosmolarity could set in patients with COVID-19 (Aloysius et al., 2020; Ren et al., 2020; Rubino et al., 2020). On the other hand, worse consequences are observed in COVID-19 patients presented with diabetes, bracketed together with a range of associated conditions augmenting the risk (Apicella et al., 2020). Hyper and hypoglycemia could both be projected as plausible predictors of mortality in critically ill individuals in COVID-19 (Codo et al., 2020; Piarulli and Lapolla, 2020). In the context of the afore-stated biunivocal link between COVID-19 and diabetes, CRISPR-Cas tools hold the potential to offer exceptional experimental strategies to elucidate the mechanisms of β-cell functions, besides the plausibility of generating gene knockouts and diabetic mouse models, amending and introducing point mutations, engineering fluorescent reporter cell lines and modulating transcription (Roh et al., 2018; Balboa et al., 2019; Khan, 2019). As a piece of exemplary evidence, Grotz et al. (2019) had reported the development of a robust pipeline to generate a stable CRISPR/Cas9 gene knockout in the human β-cell line, EndoC-βH1, to have a better insight into the genes engaged in β-cell malfunction and T2DM pathogenesis. In another report, a CRISPR/Cas9 conjugation platform was harnessed to engineer INS-1E β-cells with an objective to repurpose the insulin-releasing biomachinery, leading to glucose-dependent release of IL-10, an immunomodulatory factor (Lim et al., 2020). CRISPR/Cas9-engineered β-cells have also been explored to characterize the pharmacology of compounds with activity at both glucose-dependent insulinotropic polypeptide receptor (GIPR) and glucagon-like peptide-1 receptor (GLP-1R) (Naylor et al., 2016). Similarly, injection of lecithin based nanoliposomal carrier of CRISPR/Cas9 directly into type 2 diabetes mellitus (T2DM) db/db mice was reported to disrupt expression of dipeptidyl peptidase-4 (DPP-4) gene, consequently lowering the DPP-4 enzyme activity, complemented by normalized blood glucose levels, insulin response, and lessened organ destruction (Cho et al., 2019). In another pertinent work, Maxwell et al. (2020) had resorted to CRISPR/Cas system to rectify a Wolfram syndrome 1 (*WFS1*) (characterized by a gamut of symptoms including diabetes, optic atrophy, etc.) pathogenic variant in patient fibrobrast-derived induced pluripotent stem cells (iPSCs), followed by six-stage differentiation to pancreatic β-cells. The amended SC- β-cells exhibited up-scaled insulin secretion, dip in the expression of genes linked to endoplasmic reticulum stress as well as the reversal of hyperglycemia for half-a-year post-transplantation into diabetic mice. These studies obviously raise hopes and anticipations in harnessing CRISPR/Cas tools to address diabetic status, particularly in the milieu of the growing evidence of the link between diabetes and COVID-19. Nevertheless, the readers are suggested to peruse the recent retrospect article by Balboa et al. (2019) on the prospects and challenges of genome editing of human pancreatic β-cell models.

On a concluding note, a recent article by Soni (2020), published in *The Conversation* merits special mention. Post-emulating the plausibility of using CRISPR to (a) edit and inactivate the SARS-CoV-2 genome and (b) develop COVID-19 diagnostic platforms, the write-up is winded up with a question, germane to the context: ‘*In case, we fail to contain the virus, would/should we resort to CRISPR/Cas based gene editing in humans to possibly nullify the chances of getting infected?*’ In this regard, the ethical and legal considerations pertaining to CRISPR based genome editing research must be acknowledged and prudently deliberated in the process of determining the application of the technology (Niemiec and Howard, 2020). Particularly, in the case of at-home application, researchers are apprehensive about the plausible ethical challenges with respect to data sharing/patient privacy and issues pertaining to the requirement for befitting clinical counseling post-obtaining of the test results (Bolt, 2019). Nevertheless, the development of facile, robust, high accuracy, sensitive, affordable and point-of-care diagnostics for early detection of SARS-CoV-2 virus at home or in small clinics is the need of the hour to expedite intervention and treatment as well as to thwart the spread of COVID-19. It is highly anticipated that biotechnological advancements and concerted efforts of various researchers would surely open the avenue to a world free from the clutches of COVID-19 (*and alike*).

## Author Contributions

RK reviewed the literature, critically analyzed it, and drafted the manuscript.

## Conflict of Interest

The author declares that the research was conducted in the absence of any commercial or financial relationships that could be construed as a potential conflict of interest.
